# Therapeutic Updates for Relapsed and Refractory Classical Hodgkin Lymphoma

**DOI:** 10.3390/cancers12102887

**Published:** 2020-10-08

**Authors:** Timothy J Voorhees, Anne W Beaven

**Affiliations:** Department of Internal Medicine, Division of Hematology, Lineberger Comprehensive Cancer Center, University of North Carolina, Chapel Hill, NC 27514, USA; Timothy.Voorhees@unchealth.unc.edu

**Keywords:** Hodgkin lymphoma, brentuximab vedotin, nivolumab, pembrolizumab, salvage therapy, novel therapy, CD30 CAR-T

## Abstract

**Simple Summary:**

The approach to relapsed and refractory classical Hodgkin lymphoma is rapidly evolving. Over the past five years, we have seen the arrival of novel immunotherapies with impressive clinical response rates. These agents are actively being investigated earlier in the sequence of therapies and in combination with other clinically active therapies. We are also witnessing the arrival of new therapies for multiply relapsed disease such as chimeric antigen receptor T-cell therapy, which holds immense promise for patients in the future. This review aims to provide an overview of recent updates to both the standard of care and investigational approaches for patients with relapsed and refractory classical Hodgkin lymphoma.

**Abstract:**

Hodgkin lymphoma (HL) is a B-cell malignancy representing approximately one in ten lymphomas diagnosed in the United States annually. The majority of patients with HL can be cured with chemotherapy; however, 5–10% will have refractory disease to front-line therapy and 10–30% will relapse. For those with relapsed or refractory (r/r) HL, salvage chemotherapy followed by autologous stem cell transplant (ASCT) is standard of care, but half of patients will subsequently have disease progression. Relapse following ASCT has been associated with exceedingly poor prognosis with a median survival of only 26 months. However, in recent years, novel agents including brentuximab vedotin (BV) and programmed cell death protein 1 monoclonal antibodies (anti-PD-1, nivolumab and pembrolizumab) have been shown to extend overall survival in r/r HL. With the success of novel agents in relapsed disease after ASCT, these therapies are beginning to show clinically meaningful response rates prior to ASCT. Finally, a new investigation in r/r HL continues to produce promising treatment options even after ASCT including CD30 directed chimeric antigen receptor T-cell therapy. In this review, we will discuss the recent advances of BV and anti-PD-1 therapy prior to ASCT, novel approaches in r/r HL after ASCT, and review active clinical trials.

## 1. Introduction

Hodgkin lymphoma (HL) is a B-cell malignancy, representing approximately 10% of all lymphomas seen in the United States with an incidence of over 8500 cases per year [1]. Cases typically present with a bimodal age distribution with a peak in new cases between ages 21 and 30 years and a second peak in new cases over 60 years [2]. Classical HL (cHL) is characterized by Reed–Sternberg cells with an immunophenotype of CD15+, CD30+, CD20−, and CD45− and a tumor microenvironment marked by an extensive immunosuppressive immune infiltrate [3].

The majority of patients with either limited stage or advanced-stage cHL can be cured with chemotherapy; however, 5–10% will have refractory disease to frontline therapy and approximately 10–30% will relapse [4]. Historically, the standard of care for patients with relapsed/refractory (r/r) disease is salvage chemotherapy followed by autologous stem cell transplantation (ASCT). However, with the advent of novel agents, including brentuximab vedotin (BV) and programmed cell death protein 1 monoclonal antibodies (anti-PD-1, nivolumab and pembrolizumab), the treatment landscape for r/r HL is rapidly changing [5,6]. In this review, we will focus on recent advances in therapy options for patients with r/r cHL.

## 2. Approach to r/r cHL Following Front-Line Therapy

The approach to r/r cHL has involved high-dose chemotherapy and ASCT for over thirty years. In 1993, Linch et al. first compared the clinical responses in patients treated with mini-BEAM (carmustine, etoposide, cytarabine, melphalan) to patients receiving full dose BEAM plus ASCT. While there was no overall survival benefit, there was improvement in progression-free survival (PFS) favoring BEAM plus ASCT [7]. Similar results were seen in a second randomized study comparing dexamethasone plus BEAM to BEAM plus ASCT [8]. The improved outcomes with ASCT led it to become the standard of care approach for r/r cHL.

Multiple prognostic models have been developed to predict the outcome of patients with r/r cHL undergoing ASCT. Adverse prognostic factors have included: end of first-line chemotherapy to relapse <12 months, primary refractory disease, number of prior lines of therapy, time from diagnosis to ASCT, extranodal disease, clinical stage, anemia, and B symptoms at relapse [9,10,11,12,13,14,15]. Subsequent studies have shown the importance of having chemosensitive disease prior to ASCT. Moskowitz et al. reported a 10 year overall survival (OS) difference of 66% vs. 17% in those with chemosensitive versus chemorefractory disease prior to ASCT [16]. Further proving the importance of depth of response prior to ASCT, Sirohi et al. compared a variety second-line treatment approaches achieving a complete response (CR), partial response (PR), or chemorefractory disease prior to ASCT, showing a 10 year OS of 72%, 54%, and 11%, respectively [17].

### 2.1. Traditional Chemotherapy Based Salvage Approaches

Until recently, the only option available to achieve a CR prior to ASCT was cytotoxic chemotherapy. The most commonly utilized salvage chemotherapy regimens include DHAP (cytarabine, cisplatin, dexamethasone) and ICE (ifosfamide, carboplatin, etoposide), which have been associated with CR rates of only 21–26%, and objective response rates (ORR) of 88%, (Table 1) [17,18]. In a retrospective analysis of patients with either r/r non-Hodgkin lymphoma (NHL) or cHL, ICE appeared to have a marginally superior CR rate and ORR; however, these regimens have not been directly compared [19].

Many other salvage chemotherapy regimens have since been evaluated with the goal of improving the rate of CR prior to ASCT. GVD (gemcitabine, vinorelbine, liposomal doxorubicin) was associated with a CR rate of 19% and is infrequently used as salvage [20]. IGEV (ifosfamide, gemcitabine, vinorelbine, prednisolone) and ESHAP (etoposide, cisplatin, cytarabine, methylprednisolone) both showed an improvement in the rate of CR, 54% and 50% [21,22]; however, they failed to improve PFS following ASCT. Comparing 3–4 year PFS in those treated with ICE, DHAP, GVD, IGEV, or ESHAP reveals fairly consistent PFS estimates ranging from 52–59%, (Table 1). Given the similar outcomes, these regimens are all reasonable options for chemotherapy based salvage prior to ASCT.

BEGEV (gemcitabine, vinorelbine, bendamustine, prednisone) has also shown very encouraging results as well with a reported CR rate of 75% and ORR of 83%. Nearly ninety percent of patients proceeded to ASCT with an impressive 5-year PFS estimate of 77%, (Table 1) [23,24]. Furthermore, this regimen was well tolerated with grade 3 or higher adverse events limited mainly to hematologic toxicity (14%) and infection (7%). The high response rate and durable PFS benefit following ASCT have led this to become another acceptable standard of care option for second-line therapy of r/r cHL. Despite encouraging results with newer cytotoxic regimens such as BEGEV, novel approaches are still needed for patients with r/r cHL to further improve outcomes.

### 2.2. Novel Salvage Approaches

The past decade has shown the emergence of several exciting therapy options for r/r cHL. Brentuximab vedotin, an antibody-drug conjugate targeting the CD30 molecule, and anti-PD-1 monoclonal antibodies (nivolumab and pembrolizumab) have changed the landscape of r/r cHL following ASCT with high clinical response rates while maintaining a relatively tolerable side effect profile. These agents have recently been studied as second-line therapy options prior to ASCT.

Brentuximab has shown efficacy in first relapse when given as a single agent or when combined with other agents. In a non-randomized phase II study of sixty-five patients, BV was administered weekly for 3 weeks on a 28 day cycle at a dose of 1.2 mg/kg (note the different dose and schedule from the approved dose after ASCT). After two cycles, patients underwent disease assessment and either proceeded to ASCT consolidation if in a CR or subsequent augmented ICE prior to ASCT if ≤ PR. Eighteen patients (28%) treated with BV monotherapy achieved a CR after two cycles and proceeded directly to ASCT. The 6 year PFS estimate in this group was 80%, (Table 2) [25,26]. The remaining 47 patients with a positive PET after BV received augmented ICE salvage with an additional 35 (54%) patients achieving a CR prior to ASCT. The 6 year PFS estimate in those treated with BV and augmented ICE followed by ASCT was 82%. Both BV monotherapy and BV plus augmented ICE followed by ASCT resulted in higher 6 year PFS estimates as compared to most standard chemotherapy approaches previously described.

Multiple trials have studied the combination of BV with chemotherapy such as DHAP, ICE or bendamustine with high response rates [27,28,29]. However, due to its excellent toxicity profile, the BV-bendamustine regimen is the most widely used in clinical practice. Sixty-four patients with r/r cHL were enrolled in a phase I/II study of this combination as second-line therapy prior to ASCT. The rate of CR was 32% with an ORR of 71% [29]. BV-bendamustine resulted in a 2 year PFS estimate of 70% in those who underwent ASCT and 63% in the whole series, (Table 2). Importantly, this was a well tolerated regimen with grade 3 or higher toxicities limited to hematologic toxicity in 35% and lung infection in 14%.

Nivolumab has also been studied as a single agent prior to ASCT and resulted in an ORR of 89% and a CR rate of 59%. Approximately 75% of patients treated with nivolumab went on to complete ASCT with a 1 year PFS estimate of 79%, (Table 2) [30]. Anti-PD-1 antibody therapy is also being studied in combination with chemotherapy. In a small study of pembrolizumab-GVD, the CR rate was 93% and ORR was 100% [31]. All patients underwent ASCT, but longer follow up and a larger patient cohort will be needed to fully characterize these responses.

There is significant interest in combining non-cytotoxic agents in hopes of increasing both efficacy and tolerability. Perhaps the most promising combination at this time is BV-nivolumab prior to ASCT. This was recently studied in a single arm phase I/II study resulting in a high CR rate of 67% and an ORR 85%. The 2 year PFS estimate in those who completed ASCT was 91% and 78% in the whole series, (Table 2) [32,33]. Immune adverse events requiring steroids were uncommon (8%) and included two patients with colitis, one patient with elevated aspartate aminotransferase levels and three patients with pneumonitis [32,34]. Overall, this regimen had a tolerable toxicity profile.

Building on the success of BV-nivolumab, a double-arm phase II study is currently recruiting patients with r/r cHL to treatment with either BV-nivolumab or BV-nivolumab plus ipilimumab (CTLA-4 antibody), NCT01896999. Phase I results showed a high clinical response rate in the triplet therapy group with a CR rate of 73% and ORR 82%; however, triplet therapy was also associated with grade 3–4 treatment-related adverse events in 50% of patients [35,36]. With increased toxicity, the benefit of high clinical response rates is not yet known. However, the high response rates are very encouraging and may provide a chemotherapy-free salvage approach in the future.

While chemotherapy continues to have an important role in salvage therapy prior to ASCT for r/r cHL, novel targeted therapy and immune activating agents are quickly proving to induce high clinical response rates. Incorporation of these approaches into salvage therapy for high-risk patients, such as those with chemorefractory disease and relapses <12 months from the end of first-line therapy, may be of particular benefit as this cohort has a clear need for more effective approaches. At this time, single-agent BV, BV-bendamustine, or BV-nivolumab are all considered standard of care options for second-line therapy of r/r cHL, but in the coming years some of the other options such as single-agent anti-PD-1 antibodies or triple therapy with BV-nivolumab-ipilimumab may be added to the armamentarium.

### 2.3. Maintenance Therapy after ASCT

ASCT is curative for many patients with r/r cHL. However, patients with high-risk disease continue to frequently relapse after transplant with poor long-term prognosis. Fortunately, maintenance BV after transplant has been shown to significantly improve outcomes for this high risk group.

In the randomized, phase III AETHERA trial, high risk was defined as primary refractory disease, relapse <12 months from the end of first-line therapy, or extranodal disease at the time of relapse prior to ASCT. Of note, all patients were BV naïve at the time of enrollment. The 5 year PFS estimates were 59% in the ASCT plus BV maintenance group and 41% in the ASCT plus placebo group [37,38]. This PFS benefit came at the expense of 67% of patients developing any grade peripheral neuropathy and 13% experiencing ≥ grade 3 neuropathy. Eighty-five percent of patients who developed peripheral neuropathy did experience resolution or improvement in symptoms. An overall survival benefit has not yet been proven. While this is an option following ASCT for high-risk disease, as more patients receive BV as part of salvage therapy, the role of BV maintenance may become less clear. An expert panel recently addressed this question with universal support to continue recommending maintenance BV for patients with limited prior BV exposure (~4–6 cycles) who did not have BV refractory disease, grade C recommendation (expert opinion) [39].

Anti-PD-1 therapy has also been studied in maintenance following ASCT. Pembrolizumab was evaluated in 30 patients and 90% had a high risk of relapse. Twenty-eight patients entered this study with a CR after ASCT and two patients were in a PR. Toxicity was limited to grade 3 or higher immune-related adverse events in 20% of patients. The 2 year PFS estimate was 82%. This study provides rationale for anti-PD-1 therapy as maintenance following ASCT, though without a comparison arm, this is not currently a standard recommendation.

## 3. Approach to r/r cHL with Relapse after ASCT or Ineligible for ASCT

Historically, outcomes for patients who were refractory to salvage chemotherapy or relapsed after ASCT have been abysmal. These patients had few effective treatment options and little chance of long-term remission except potentially with an allogeneic stem cell transplant in a select group of patients. The median overall survival for patients with relapsed cHL after ASCT has been reported between 12–24 months [5]. Fortunately, the past ten years has shown major advances in the treatment of r/r cHL with Food and Drug Administration (FDA) approval of three novel agents: BV, nivolumab, and pembrolizumab. In the relapsed setting, the use of novel agents has been associated with improved survival compared to traditional cytotoxic options with a median survival of 86 versus 17 months [6].

### FDA Approved Therapies for Multiply r/r cHL

Brentuximab vedotin was the first agent FDA approved for r/r cHL after ASCT or following two lines of therapy. This was based on results of a phase II study in 102 patients with r/r cHL after ASCT with a median of 3.5 prior chemotherapy regimens not including ASCT. Patients received BV at a dose of 1.8 mg/kg every 3 weeks for up to 16 cycles with an impressive ORR of 75% and a CR rate of 34% [40]. Although the median PFS benefit was only 5.6 months, results in patients with a CR were very durable with an estimated 5 year PFS of 52% as compared to 22% in the whole series. Furthermore, 26% of all patients who achieved a CR (*n* = 9) have had durable remissions with no further therapy after stopping BV. This suggests that there may be a small group of patient who are cured, or at least have very durable remissions, with BV alone [41]. However, treatment with BV was also associated with peripheral neuropathy in 55%, grade 3 peripheral neuropathy in 11%, and grade 3–4 neutropenia in 20%. While 80% had improvement in peripheral neuropathy, only 50% had resolution of neuropathy symptoms after stopping treatment.

Nivolumab was the first anti-PD-1 antibody therapy evaluated in a phase II study (CheckMate 205) at a dose of 3 mg/kg every 2 weeks until disease progression or unacceptable toxicity [42,43]. A total of 243 patients were treated with r/r disease after ASCT. This was a heavily pretreated group with a median of four prior lines of therapy, which included prior BV in 74%. The ORR was 69% and the CR rate was 16% with a median PFS of 15 months. Responses were slightly higher in the BV naïve cohort with a CR rate of 29% and median PFS of 18 months [44]. The most common immune-related adverse events were hypothyroidism (12%), rash (11%), hepatitis (9%), pneumonitis (4%), and colitis (4%), though the majority spontaneously resolved without the need for steroid administration. The FDA has approved nivolumab in r/r cHL following ASCT and BV, or at least three prior lines of therapy, at doses of 240 mg every 2 weeks or 480 mg every 4 weeks.

Pembrolizumab was the second anti-PD-1 antibody therapy studied in a phase II study (KEYNOTE-087) at a dose of 200 mg every 3 weeks. This study included subjects who had progression after ASCT and subsequent BV, progression with chemotherapy and BV prior to ASCT, and progression after ASCT but BV naïve. A total of 210 patients were treated resulting in an ORR of 69% and CR rate 22% [45,46]. Extended follow up from KEYNOTE-087 demonstrated a median PFS of 14 months with the best PFS outcomes in those with progression after ASCT but BV naïve (median PFS 19 months) [47]. The safety profile of pembrolizumab was very similar to nivolumab with infrequent immune adverse events. The FDA has approved pembrolizumab in r/r cHL following three or more lines of therapy at doses of 200 mg every 3 weeks or 400 mg every 6 weeks.

In the first direct comparison trial of novel agents in r/r cHL, Kuruvilla et al. recently reported results from KEYNOTE-204, a randomized phase III study of pembrolizumab versus BV. Patients were randomized and stratified by prior ASCT, primary refractory disease, and relapse less than or greater than 12 months from end of first-line therapy. This study included 300 patients. The ORR of pembrolizumab was 66% versus 54% with BV. Complete responses were observed in 24% of each group. The median PFS was 12.6 months with pembrolizumab and 8.2 months with BV and the median duration of response was 21 months with pembrolizumab and 14 months with BV [48]. This study supported the superiority of pembrolizumab (anti-PD-1 therapy) over BV in r/r cHL; however, there are still few long-term, relapse-free survivors in either treatment group, so most patients will eventually be treated with both agents at some time in the course of therapy for r/r cHL. A second randomized phase III study of BV versus BV-nivolumab is currently ongoing in patients with r/r cHL following ASCT or those ineligible for ASCT, NCT03138499.

Critical to the appropriate use of immune activating therapies such as anti-PD-1 and CTLA-4 antibody therapies is the understanding of possible pseudoprogression. Disease assessments for conventional chemotherapy regimens in lymphoma are based on the Lugano criteria, which generally measure the size of lesions as well as Deauville score from positron emission tomography (PET) [49]. Pseudoprogression is a radiologic phenomenon in which therapeutic immune activation manifests as either an increase in the size of lesions or appearance of new lesions without true progressive disease. To address this issue, the lymphoma response to immunomodulatory therapy criteria (LYRIC) were created in 2016, which added the category indeterminate responses (IR) to the traditional Lugano response criteria [50]. While there is insufficient prospective analysis of IR responses, the committee recommends continuing therapy for any IR response and repeating PET imaging to confirm either ongoing response or disease progression. It is imperative for treating physicians to understand the LYRIC criteria in order to avoid stopping an effective drug too early.

The optimal duration of anti-PD-1 therapy in patients who are fortunate to have long-term responses is not known. In the phase II study of nivolumab in r/r cHL, patients received therapy until disease progression or unacceptable toxicity [43]. Extended follow up revealed that, at a median follow up of 18 months, 40% of patients remained on therapy [44]. In the phase II study of pembrolizumab, patients received therapy until disease progression or a maximum of 24 months [46]. Three-year follow up from this study revealed that 22% of patients completed the 24 months of therapy [47]. While there is no data to provide a recommendation for stopping anti-PD-1 therapy at a specific timepoint, most clinicians will have an informed discussion with patients in remission as they approach 24 months of therapy regarding the risks and benefits of stopping anti-PD-1 therapy.

The high response rates with promising duration of response seen with new agents such as BV and anti-PD-1 after ASCT are very exciting. There is ongoing work investigating whether these drugs can be effective as multi-drug combinations or administered earlier in the disease course.

## 4. Approach to Multiply Relapsed cHL

### 4.1. Retreatment with Brentuximab Vedotin and Anti-PD-1 Therapy

The management of r/r cHL has taken a significant step forward over the past decade. The pivotal studies that have resulted in FDA approval of BV, nivolumab, and pembrolizumab have previously been discussed. As more patients receive these drugs as first or second-line therapy, their utility as part of later lines is unclear. However, there is increasing data that some patients can have durable remissions with retreatment at disease progression.

Outcomes for twenty-one patients who were retreated with BV after a median of 11.4 months since stopping the drug, were excellent. The ORR in this group was 60% with a CR rate of 30% [51]. Additionally, of sixteen patients from KEYNOTE-087 who stopped therapy after achieving a CR, 11 (69%) were able to achieve an objective response when re-challenged with pembrolizumab at relapse, and 5 (31%) patients achieved a second CR [47]. In a small real-world subset of patients who discontinued either nivolumab or pembrolizumab while in a CR, five of seven patients were able to achieve a second CR with re-challenge of the same anti-PD-1 agent [52]. Investigation into this phenomena is needed, especially as these therapies are administered earlier in treatment sequence. Furthermore, evaluating how interval therapy may or may not impact the outcome of retreatment with BV or anti-PD-1 therapies is an unanswered question at this time.

### 4.2. Other Agents for Multiply Relapsed Disease

Therapy options in r/r cHL following ASCT, BV, and anti-PD-1 therapy include a variety of less intensive chemotherapy approaches and several targeted or immunomodulatory agents. Bendamustine has been shown to have clinical activity as a single agent (ORR 53%, CR 25%) as well as in combination with carboplatin and etoposide [53,54]. Gemcitabine-based regimens including GemOx (gemcitabine, oxaliplatin) and GCD (gemcitabine, carboplatin, dexamethasone) have both been shown to have clinical activity [55,56]. Unfortunately, responses to chemotherapy in multiply relapsed cHL are usually short.

Based on preclinical work supporting the activation of the AKT-mTOR pathway in cHL, the mTOR inhibitor everolimus was studied in a phase II study of 19 patients at a dose of 10 mg daily. The ORR was 47%, which consisted of mostly partial responses and only one CR. The median PFS was only 6 months [57]. In a larger study of 57 patients treated with everolimus, the ORR was 42%, and 9% achieved a CR. Approximately 12% of patients were able to remain on everolimus for over 3 years [58]. This has not resulted in an FDA approval for everolimus in the treatment of r/r cHL; however, given that everolimus is very well tolerated, this is a reasonable consideration in multiply relapsed cHL or in a patient who may be frail and unable to tolerate other therapies.

Lenalidomide is an immunomodulatory agent which is thought to induce apoptosis of malignant B cells through interaction with ubiquitin E3 ligase and degradation of Ikaros transcription factors. It is also believed to alter tumor microenvironments and promote T-cell-mediated immune responses. Lenalidomide has been studied in a single-arm phase II study at a dose of 25 mg daily for 21 of 28 days. The ORR was 19% with only 1 CR, though a cytostatic ORR (CR, PR and stable disease > 6 months) was 33% [59]. Correlative work from this study revealed that the responders in this study had a significant decrease in CCL22 levels in the peripheral blood. This chemokine is known to interact with CCR4 receptors, which is often found on Tregs as well as a variety of other immune cells, suggesting a role for microenvironment modulation. This could be of interest in the future for assessing sequential therapy with anti-PD-1 agents. While lenalidomide is not FDA approved for r/r cHL, it is an acceptable option for patients with multiply relapsed disease.

### 4.3. Role for Allogeneic Stem Cell Transplantation (alloSCT)

AlloSCT still deserves discussion for patients with multiply r/r cHL. Historically, patients treated with myeloablative conditioning prior to alloSCT experienced prohibitively high non-relapse mortality approaching 50% at 5 years [60]. As the importance of the graft versus lymphoma effect in alloSCT became more apparent, reduced intensity conditioning (RIC) regimens began to show significantly lower non-relapse mortality and improved 3 year PFS and OS estimates at 42% and 56%, respectively [61,62]. Although, in a meta-analysis of 38 separate reports of alloSCT for r/r HL, there was a steady decline in relapse-free survival and OS until 3 years of follow up, without a clear plateau of survival [63]. Even in the modern era of managing r/r cHL with the ability to obtain prolonged remissions and disease control with novel agents such as BV and anti-PD-1 therapies, there will still remain a small group of highly refractory patients for whom this is the only potentially curative option.

Adding to the complexity of alloSCT in r/r cHL, new data suggest that anti-PD-1 therapy both before and after alloSCT increase toxicity of alloSCT. Anti-PD-1 therapy used immediately prior to alloSCT has been linked to increased risk of veno-occlusive disease and severe graft versus host disease. Furthermore, there are known risks of acute and chronic graft versus host disease occurring with anti-PD-1 therapy administered after alloSCT [64,65,66]. In a recent retrospective study comparing patients with or without anti-PD-1 exposure immediately prior to haploidentical alloSCT, patients with anti-PD-1 exposure had a trend towards lower 2 year relapse-free survival, though long-term follow up is limited in the cohort [67]. For a young, otherwise healthy patient with r/r cHL after ASCT, a thorough discussion regarding the risks and possible benefits of alloSCT is worthwhile, though the ideal timing and role of alloSCT remains unclear.

### 4.4. Investigational Approaches

There are a variety of experimental therapies being developed for r/r cHL at this time. Perhaps the most advanced novel therapy option for r/r cHL is chimeric antigen receptor (CAR) T-cell therapy specific for CD30 (CD30.CAR-T). Ramos et al. reported the results of a phase I study of CD30.CAR-T in 2017, proving safety but few clinical responses [68]. Importantly, these patients were not treated with lymphodepletion prior to CD30.CAR-T infusion. In two separate parallel phase I/II studies in which patients were treated with lymphodepletion prior to CD30.CAR-T, the ORR in 32 patients with active disease who received fludarabine-based lymphodepletion was 72% including a CR rate of 59%, (Table 3). Importantly, CD30.CAR-T was well tolerated with Grade 3 or higher toxicity limited to hematologic toxicity. Grade 1 cytokine release syndrome was observed in 24% of patients [69]. The vast majority of patients had prior ASCT, BV, and anti-PD-1 therapy. The 1 year PFS estimate was 36%. Many patients who progressed following CD30.CAR-T were re-challenged with anti-PD-1 therapy with encouraging clinical responses [70]. A prospective pilot study evaluating the clinical activity and immunomodulatory effect of re-challenging with anti-PD-1 therapy after CD30.CAR-T progression is currently accruing, NCT04134325. Additionally, a multi-site pivotal phase II trial of CD30.CAR-T in r/r cHL is planned to start accrual near the end of 2020, NCT04268706.

A second phase I/II trial is actively accruing patients with r/r cHL to treatment with a CD30 and CCR4 co-expressing CAR-T cell (CD30.CCR4.CAR-T), NCT03602157. This novel CAR-T contains the CD30.CAR-T construct previously studied followed by an internal ribosomal entry site domain and a CCR4 gene. This results in co-expression of both molecules (CD30.CAR and CCR4) on the cell surface of the CD30.CCR4.CAR-T. The rationale of overexpressing CCR4 is to allow for CAR-T migration and homing to the tumor microenvironment. In cHL specifically, TARC is known to be secreted in a high quantity from Reed–Sternberg (RS) cells, which can result in Treg migration to the tumor microenvironment through the CCR4 expression on Tregs [71,72]. This approach has been evaluated in a mouse model of cHL, which showed enhanced tumor killing by CD30.CCR4.CAR-T cells as compared to CD30.CAR-T cells [73]. The CCR4 overexpression on CD30.CCR4.CAR-T cells aims to exploit this tumor physiology to allow for CD30.CCR4.CAR-T cells to better home to sites of disease.

A bispecific, tetravalent chimeric antibody targeting CD30 and CD16, AFM13, has also been evaluated in r/r cHL. This bispecific antibody is designed to target CD30 on RS cells in cHL and activate NK cells through CD16. In the phase I study, 12% of patients achieved a PR, and 50% had stable disease, (Table 3) [74]. In a follow up Phase Ib study in anti-PD-1 naïve patients, AFM13 used in combination with pembrolizumab resulted in an ORR of 87% and CR rate of 39% [75]. Additionally, a phase I trial is being developed investigating AFM13 in combination with allogeneic off-the-shelf umbilical cord blood-derived NK cells pre-loaded with AFM13, NCT04074746.

Epigenetic modification with histone deacetylase inhibitors (HDACi) and hypomethylating agents (HMA) have the potential to result in tumor immunomodulation with a priming effect prior to anti-PD-1 therapy. In patients with r/r cHL, both HDACi and HMA have been studied as monotherapy and in combination with anti-PD-1 therapy. In a phase II study of patients with relapse after ASCT, 129 patients were treated with panobinostat, a potent pan-deacetylase inhibitor, with an ORR of 27%, CR rate of 4%, and a median PFS of 6 months, (Table 3) [76]. Vorinostat, a class I/II/IV HDACi, is currently being studied in combination with pembrolizumab in a phase I study enrolling multiple B-cell lymphomas, NCT03150329. Nie et al. reported results of a two-arm phase II study with randomization to either camrelizumab monotherapy (anti-PD-1 antibody) or camrelizumab plus decitabine (HMA) in r/r, anti-PD-1 naïve, cHL. Camrelizumab plus decitabine resulted in an ORR of 95%, CR rate 71%, and 1 a year PFS of 89% [77]. Both HDACi and HMA have promise when combined with immunotherapy, though further investigation of the clinical benefit and understanding of their immunomodulatory effects is needed.

Agents targeting B-cell receptor signaling have also been evaluated in r/r cHL. Bruton’s tyrosine kinase (BTK) expression has been seen in 22% of patients with cHL [78]. Several case reports of remarkable response to the BTK inhibitor ibrutinib have been reported after alloSCT for r/r cHL [79]. Ibrutinib is currently being evaluated as monotherapy in a phase II study, NCT02824029, and in combination with nivolumab or BV in two additional studies, NCT02940301 and NCT02744612. Additionally, anti-LAG-3 antibodies are also being investigated to target exhausted T-cells, which have been shown to be present in the tumor microenvironment of patients with cHL. In a phase I/II study, the anti-LAG-3 antibody BMS-986016 is being studied alone and in combination with nivolumab, NCT02061761.

The future of novel approaches to r/r cHL likely include a variety of sequential therapy options that not only target the immune microenvironment, but attempt to modulate the tumor microenvironment allowing for both endogenous and therapeutic immune cell targeting.

## 5. Conclusions

The past five years have shown a dramatic change in the approach to r/r cHL, moving away from multi-agent chemotherapy regimens towards targeted therapy and immunotherapies. The approach to salvage therapy prior to ASCT is changing rapidly and may eventually bypass cytotoxic chemotherapy altogether. In the next five years, as increasing data become available, we will learn more about how best to utilize these novel agents and begin to incorporate new investigational agents such as CAR-T cell therapy in the management of r/r cHL. The ongoing role of ASCT as consolidation following salvage therapy with BV, anti-PD-1 therapy, or combination therapies will need to be investigated. The lack of a clear standard of care approach for salvage therapy continues to result in issues with trial design to assess the value of ASCT after salvage therapy. For now, ASCT following salvage therapy remains the community standard of care approach. Finally, the sequence of therapies and impact of interval therapies on the ability to pursue retreatment with novel agents will be an important area of investigation. As more highly active agents become available earlier in the treatment course, determination of a recommended treatment pathway for specific patient populations with r/r cHL will be needed.

## Figures and Tables

**Table 1:** Salvage chemotherapy options for relapsed or refractory (r/r) classical Hodgkin lymphoma.

Agent	Dose/Schedule		Patients Evaluable	CR/ORR	% ASCT	PFS	Median Follow up
ICE	Etoposide 100 mg/m^2^ Day 1–3	Every 3 weeks	65	26%/88%	88%	58% ^‡^	43 months
	Ifosfamide 5 g/m^2^ Day 2						
	Carboplatin AUC 5 Day 2						
DHAP	Cisplatin 100 mg/m^2^ Day 1	Every 3 weeks	102	21%/88%	100%	59% ^†^	30 months
	Cytarabine 2 mg/m^2^ q12hr Day 2						
	Dexamethasone 40 mg Day 1–4						
GVD	Gemcitabine 1000 mg/m^2^ Day 1 and 8	Every 3 weeks	41	19%/70%	95%	52% ^†^ (4 year)	43 months
	Vinorelbine 20 mg/m^2^ Day 1 and 8						
	Doxorubicin 15 mg/m^2^ Day 1 and 8						
IGEV	Ifosfamide 2000 mg/m^2^ Days 1–4	Every 3 weeks	91	54%/81%	86%	53% ^†^ (3 year)	26 months
	Gemcitabine 800 mg/m^2^ Days 1 and 4						
	Vinorelbine 20 mg/m^2^ Day 1						
	Prednisolone 100 mg Days 1–4						
ESHAP	Etoposide 40 mg/m^2^ Days 1–4	Every 3 or 4 weeks	82	50%/67%	91%	56 months ^†^ (median) 52 months ^‡^ (median)	87 months
	Cisplatin 25 mg/m^2^ Days 1–4						
	Cytarabine 2000 mg/m^2^ Day 5						
	Methylprednisolone 500 mg Days 1–4						
BEGEV	Gemcitabine 800 mg/m^2^ Day 1 and 4	Every 3 weeks	58	75%/83%	88%	77% ^†^/59% ^‡^	60 months
	Vinorelbine 20 mg/m^2^ Day 1						
	Bendamustine 90 mg/m^2^ Day 2–3						
	Prednisone 100 mg Day 1–4						

CR, complete response; ORR, objective response rate; ASCT, autologous stem cell transplant; PFS, progression-free survival. ^†^ ASCT; ^‡^ All patients.

**Table 2:** Novel and investigational salvage approaches for r/r classical Hodgkin lymphoma.

Agent	Dose and Schedule		Patients Evaluable	CR/ORR	% ASCT	PFS or TTP	Median Follow up	NCCN
								Rec
BV	BV 1.2 mg/kg Day 1, 8, 15 *	Every 4 weeks	65	28%/73%	26%	80% ^†^ (6 year)	72 months	Yes
BV + Benda	BV 1.8 mg/kg Day 1	Every 3 weeks	53	74%/93%	76%	70 ^†^/63% ^‡^ (2 year)	21 months	Yes
	Benda 90 mg/m^2^ Days 1–2							
BV + Nivo	Nivo 3 mg/kg Day 1	Every 3 weeks	91	67%/85%	74%	91% ^†^/78% ^‡^ (2 year)	23 months	Yes
	BV 1.8 mg/kg Day 1							
BV + DHAP	BV 1.8 mg/kg Day 1	Every 3 weeks	61	78%/87%	87%	76% ^‡^ (2 year)	21 months	No
	Cisplatin 100 mg/m^2^ Day 1							
	Cytarabine 2 g/m^2^ Day 2							
	Dexamethasone 40 mg Days 1–4							
BV + ICE	BV 1.8 mg/kg Day 1 and 8	Every 3 weeks	39	69%/92%	51%	69% ^‡^ (1 year)	N/A	No
	Ifosfamide 5 g/m^2^ Day 2							
	Etoposide 100 mg/m^2^ Day 1–3							
	Carboplatin AUC 5 Day 2							
Nivolumab	Nivo 3 mg/kg Day 1	Every 2 weeks	37	59%/89%	73%	79% ^‡^ (1 year)	11 months	No
Pembro + GVD	Pembrolizumab 200 mg Day 1	Every 3 weeks	14	93%/100%	100%	100% ^‡^	4 months	No
	Gemcitabine 1000 mg/m^2^ Day 1, 8							
	Vinorelbine 20 mg/m^2^ Day 1, 8							
	Doxorubicin 15 mg/m^2^ Day 1, 8							

CR, complete response; ORR, objective response rate; ASCT, autologous stem cell transplant; PFS, progression-free survival; TTP, time to progression; NCCN, National Comprehensive Care Network; Rec, recommended. * Not including subsequent augmented ICE; ^†^ ASCT; ^‡^ All patients.

**Table 3:** Novel investigational approaches for r/r classical Hodgkin lymphoma.

Clinical Trial ID	Trial Phase	Agent	Dose/Schedule	CR/ORR	PFS	Median Follow up
NCT02917083	Phase I/II	CD30.CAR-T	MTD: 2 × 10^8^ cells/m^2^	59%/72%	36% (1 year)	18 months
NCT02690545			Cond: Benda/Flu or Cy/Flu			
NCT02663297	Phase I	CD30.CAR-T	MTD: 2 × 10^8^ cells/m^2^	N/A	N/A	N/A
			Cond: ASCT			
NCT03602157	Phase I/II	CD30.CCR4.CAR-T	MTD under investigation	N/A	N/A	N/A
		+/− CD30.CAR-T	Cond: Benda/Flu			
NCT01221571	Phase I	CD30/CD16	MTD: 7 mg/kg	0%/12% ^†^	N/A	N/A
		bispecific antibody				
NCT02665650	Phase Ib	CD30/CD16	MTD: Pembro 200 mg + AFM13 at 7 mg/kg	35%/87%	N/A	N/A
		bispecific antibody				
		+ pembrolizumab				
NCT04074746	Phase I	AFM13-NK cells + AFM13	AFM13-NK cells Day 0	N/A	N/A	N/A
			AFM13 weekly × 4 weeks			
			Cond: Cy/Flu			
NCT03150329	Phase I	Vorinostat + pembrolizumab	MTD under investigation	NA	N/A	N/A
NCT04510610	Phase II	Decitabine + camrelizumab	Decitabine 10 mg daily, Days 1–5 + camrelizumab 200 mg, Day 8	71%/95%	89% (1 year)	15 months
NCT02824029	Phase II	Ibrutinib	560 mg daily	N/A	N/A	N/A
NCT02061761	Phase I/II	Anti-LAG-3	MTD under investigation	N/A	N/A	N/A
		+/− Nivolumab				

CR, complete response; ORR, objective response rate; PFS, progression-free survival; MTD, maximum tolerated dose. ^†^ Disease control rate 62%.
